# Effectiveness of school-based active breaks on classroom behavior, executive functions and physical fitness in children and adolescent: a systematic review

**DOI:** 10.3389/fpubh.2025.1469998

**Published:** 2025-01-30

**Authors:** Tomás Reyes-Amigo, Gabriel Salinas-Gallardo, Edgardo Mendoza, Camilo Ovalle-Fernández, Jessica Ibarra-Mora, Nicolás Gómez-Álvarez, Hernaldo Carrasco-Beltrán, Jacqueline Páez-Herrera, Juan Hurtado-Almonácid, Rodrigo Yañez-Sepúlveda, Rafael Zapata-Lamana, Felipe Sepúlveda-Figueroa, Jorge Olivares-Arancibia, Jorge Mota

**Affiliations:** ^1^Physical Activity Sciences Observatory (OCAF), Department of Physical Activity Sciences, Universidad de Playa Ancha, Valparaíso, Chile; ^2^Physical Education, Universidad Metropolitana de las Ciencias de la Educación, Ñuñoa, Chile; ^3^Physical Activity, Health and Education Research Group (AFSYE), Physical Education Pedagogy, Universidad Adventista De Chile, Chillán, Chile; ^4^Physical Education School, Pontificia Universidad Católica de Valparaíso, Viña del Mar, Chile; ^5^Faculty of Education and Social Sciences, Universidad Andres Bello, Viña del Mar, Chile; ^6^School of Kinesiology, Faculty of Health, Universidad Santo Tomás, Los Ángeles, Chile; ^7^School Education, University of Concepción, Los Ángeles, Chile; ^8^Grupo AFySE, Investigación en Actividad Física y Salud Escolar, Escuela de Pedagogía en Educación Física, Facultad de Educación, Universidad de las Américas, Santiago, Chile; ^9^Research Centre in Physical Activity, Health and Leisure, Faculty of Sports, Universidade do Porto, Porto, Portugal

**Keywords:** classroom, cognition, fitness, intervention, childhood

## Abstract

**Introduction:**

The classroom environment is ideal for promoting physical activity interventions since children spend most of their day there but often engage in sedentary behavior. Given this context, an emerging trend to promote physical activity is active breaks at school. This systematic review evaluated the effects of school-based physical activity interventions involving active breaks on children and adolescents’ classroom behavior, executive functions, and physical fitness.

**Methods:**

This review was guided by the Preferred Reporting Items for Systematic Reviews and Meta-Analyses guidelines. A literature search was conducted using PubMed, Web of Science, Scopus, and EBSCOhost. Studies published between January 2010 and August 31, 2023, including participants aged 5 to 18, were included. Interventions involving active breaks and outcomes related to classroom behavior, executive functions, and physical fitness were considered.

**Results:**

Initially, 145 studies were identified, with 22 duplicates excluded. After screening 123 articles by title and abstract, 86 were excluded. Subsequently, 37 articles underwent full-text screening, resulting in 22 included studies. Six studies showed positive effects on classroom behavior; five studies showed improvements in executive functions, and only two studies indicated increases in physical fitness.

**Discussion:**

This review suggests incorporating active breaks during school hours can improve classroom behavior in children and adolescents. However, the effects of active breaks on executive functions and physical fitness are unclear. More research is needed to fully understand the benefits of implementing active break programs in the classroom.

**Systematic review registration:**

PROSPERO, CRD42023448267, available from: https://www.crd.york.ac.uk/prospero/display_record.php?ID=CRD42023448267.

## 1 Introduction

Regular and systematic physical activity (PA) in children and adolescents is positively associated with physiological and cognitive benefits, such as cardiorespiratory fitness and executive function (1, 2). However, there are still insufficient systematic reviews that consolidate studies on the effects of active breaks (ABs) interventions on classroom behavior, executive function, and physical fitness in children and adolescents (3).

Regarding classroom behavior, a positive relationship has been reported between higher PA levels and better on-task behavior during childhood lessons, which is significant for the learning process (4). Cognitive skills are crucial for school readiness. This developmental window provides a great opportunity for PA to positively influence cognition and enrich the educational process (5).

According to the World Health Organization, children and adolescents aged 5–17 should engage in at least 60 min of moderate-to-vigorous physical activity (MVPA) daily. However, the Global Matrix 3.0 Physical Activity Report Card Grades for Children and Youth, which evaluates physical activity levels in children and youth from 49 countries, revealed a mean letter grade of C for the percentage of children and youth meeting the physical activity recommendation of 60 min of MVPA per day, representing only 27–33% of children and youth (6).

Current evidence (7) suggests that the focus and priority should be on identifying strategies to increase and maintain PA levels in children and adolescents in school, as it is one of the primary environments where they spend much of their time. A significant decrease in total daily PA was observed during the transition from primary to secondary school, highlighting the need to increase opportunities for adolescents to be physically active, particularly during this transitional period (8, 9). Contemporary school settings are key environments for promoting various PA opportunities to enhance PA participation and wellbeing. However, the school environment, including class time and after-school hours, contributes to most children’s sedentary time due to prolonged sitting and long periods of sedentary behavior during class hours (10). Consequently, current school settings may facilitate an adequate amount of PA for children and adolescents (11).

Classroom-based PA interventions involve incorporating PA during class time or between lessons with the participation of regular teachers and have emerged as a potential solution (4). In this context, classroom-based PA interventions, known as ABs, incorporate brief periods of PA into school routines and have been investigated as potential strategies to increase PA during school hours without reducing educational time or interfering with the educational process (11–14). ABs are 5–15 min sessions of moderate-to-vigorous PA intensity led by teachers who introduce short bursts of PA into academic lessons (15). ABs can be implemented in any school context because they do not require special spaces, equipment, or specialized personnel (3, 12). Moreover, a recent systematic review with a meta-analysis of the effects of AB interventions on attentional outcomes found some positive acute and chronic effects, especially on selective attention (15–17), reporting a positive impact of ABs on classroom behavior (time on task) (16) and a potential benefit on cognitive functions. Regarding the relationship between the chronic practice of ABs and the level of physical fitness and general cognitive functioning, evidence suggests that these benefits are based on increased cardiorespiratory fitness (CRF). Thus, the cognitive functional benefits associated with regular exercise are mediated by improved physical fitness. Additionally, physiological adaptations attributed to chronic PA have been linked to brain-level adaptations, which positively impact cognitive performance (18–20). Therefore, the regular practice of ABs should improve physical fitness and cognitive performance. However, existing literature on ABs shows high levels of heterogeneity in samples, intervention characteristics, and outcomes (14). In this regard, the present review aims to systematically analyze the application times, types of exercises, and study quality in ABs interventions, while presenting results for each of the study variables.

The following questions were posed to conduct this systematic review: What is the current scientific evidence regarding the effects of ABs on classroom behavior, executive function, and physical fitness in children and adolescents? What are the application times and types of exercises used in ABs? What are the effects of ABs on each variable, and how is the quality of the studies evaluated? This study aimed to evaluate the effects of school-based PA interventions based on ABs on classroom behavior, executive function, and physical fitness in children and adolescent students.

## 2 Materials and methods

### 2.1 Search strategy

This review was conducted following the Preferred Reporting Items for Systematic Reviews and Meta-Analyses (PRISMA) guidelines (21) and registered with the International Prospective Register of Systematic Reviews (PROSPERO) (registration number: CRD42023448267). The search used four electronic databases: PubMed, Web of Science, Scopus, and EBSCOhost.

The search for studies included in this systematic review was conducted between August 1 and August 31, 2023. Only studies published between January 2010 and August 31, 2023, were included as ABs have been an emerging topic of scientific interest during the last decade (22).

The search terms (MESH) and keywords used in all databases were: [active breaks OR activity break OR brain break OR classroom break] AND [children OR child OR adolescent OR schoolchildren OR students] AND [physical fitness OR physical conditioning OR cardiorespiratory fitness OR muscular fitness] AND [classroom behavior OR on-task behavior OR off-task behavior OR time-on-task] AND [cognitive function OR executive function OR executive control OR working memory OR inhibitory control OR cognitive flexibility].

### 2.2 Eligibility criteria

Studies were considered eligible if they met all of the following criteria: (a) the research involved AB interventions carried out inside the classroom (acute [single class] or chronic classes [weeks or months]), (b) the AB characteristics (e.g., the type of movements involved, intensity, work-to-recovery ratio [WRR]), and the single class or week frequency duration of the intervention were provided; (c) randomized controlled trials (RCT) and control trials (CT) were considered; (d) the research focused on different subcomponents of physical fitness (CRF, muscular fitness, flexibility, and body composition), classroom behavior (on-task behavior and time on task), and executive function (working memory, inhibitory control, and cognitive flexibility); (e) participants were aged 5–18 without diagnosed diseases or mental disorders; (f) only articles published in scientific journals were considered; (g) only research involving humans and written in English were considered.

Study protocols, articles focusing on sports, reviews, papers published in conferences, dissertations, theses, and non-peer-reviewed journals were excluded.

### 2.3 Study selection

The selection criteria were based on the PICOS criteria used to define the characteristics of the included studies (23). Population: Studies including participants aged 5 to 18 were included; Intervention with ABs: Studies evaluating the effect of acute or chronic ABs on classroom behavior, executive function, and physical fitness providing clear ABs protocols were included; Comparator: Studies comparing active control (e.g., other exercise protocols) or passive control (e.g., sitting and resting) were included; Outcomes: Studies assessing physical fitness, classroom behavior, or executive function were included; Study design: RCTs, CTs, and repeated crossover studies were included. Two authors completed the screening and selection of the studies in September 2023. First, duplicates were removed, and titles and abstracts were examined to identify studies that met the inclusion criteria. Second, the full texts of eligible studies based on the screened studies were read by three authors (TRA, EM, and FSF) to determine their final inclusion. Disagreements between the two reviewers were resolved through a consensus meeting among the three authors in September 2023. Finally, articles on acute and chronic AB interventions and their effects on physical fitness, classroom behavior, and executive function were included in this review.

### 2.4 Data extraction process and data synthesis

The full texts were analyzed, and after confirming the eligibility criteria, the following data were extracted: (a) first author’s name, publication year, and country of data collection; (b) sample size, participants’ age and sex; (c) study design and/or group assignment; (d) details about ABs intervention (such as exercise intensity, work/rest ratio, session time, frequency, duration of intervention, and modality); (e) subcomponents of physical fitness, classroom behavior, and executive functions assessed; and (f) main findings. Outcome data were extracted as pre- and postintervention means and standard deviations (SD). The dependent variables were reported in repetitions or milliseconds (if relative values were not reported). In studies that reported intermediate and post-intervention values, only the final executive function values were compared with baseline values. Data from the included studies were independently extracted by two reviewers (TRA and EM), and any discrepancies were resolved by consulting a third reviewer (FSF).

### 2.5 Methodological quality assessment

Three authors analyzed and studied the PEDro scale (24) (TRA, EM, and FSF), and then two authors (TRA and EM) assessed the study quality according to the PEDro scale for 1 week. Any disagreements were discussed with a third reviewer (FSF) until a consensus was reached. Eleven criteria of the PEDro scale were analyzed to comply with the requirements of the PRISMA protocol, as previously described (4, 17). The results were obtained using the criteria and general score for each article.

The total PEDro score was obtained by adding points describing the quality of papers, such as 9–10 (excellent), 6–8 (good), 4–5 (fair), and ≤3 (poor) (24).

Studies that are not RCT or CT were analyzed with the Risk of Bias Assessment Tool for Non-randomized Studies (RoBANS) (25).

## 3 Results

**Figure 1:**
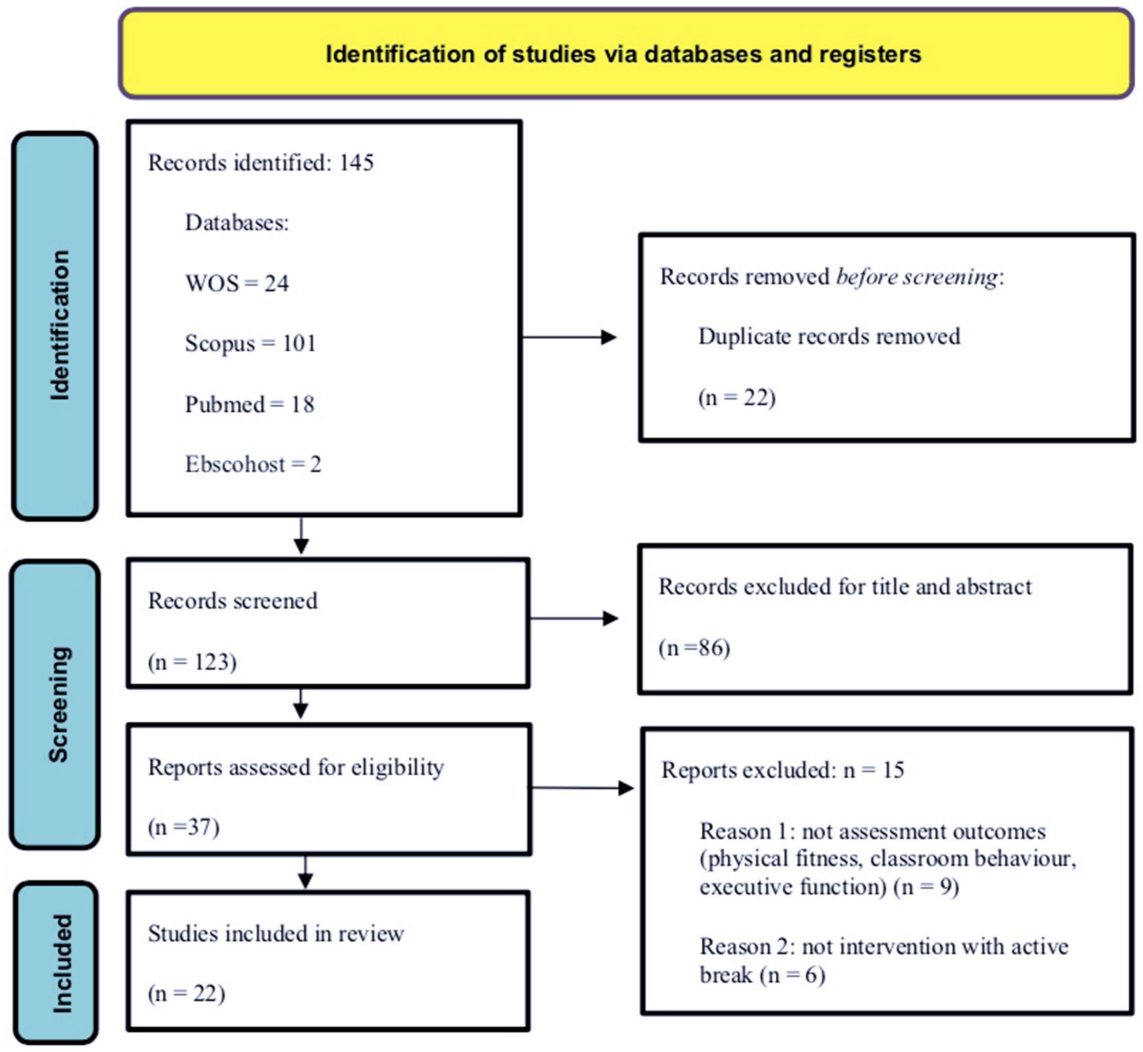
PRISMA flow diagram of each stage of the studies selection.

A total of 145 studies were initially identified in PubMed, Web of Science, Scopus, and EBSCOhost. Of these, 22 duplicates were excluded from the analysis. Accordingly, 123 articles were screened by title and abstract, of which 86 were excluded. After the first screening stage, 37 articles were selected for full-text screening, resulting in 22 full-text articles for data extraction and reporting. As the search was conducted 11 months previously, an updated review (without finding new studies according to the eligibility criteria) was performed to further refine the study findings. The flow diagram of the study selection process, following the PRISMA guidelines (21), is shown in Figure 1. Table 1 summarizes the characteristics and results of the study.

**Table 1:** Summary and description of the studies included in the review.

Authors, Country, Study Design	Sample	Groups (n)	Modality/Intensity	ABs time	Frequency	Duration	Assessments	Results
Broad et al. (2021) (26). Canada. RCT.	School Children *N* = 35; ♂ = 22; ♀ = 13 6 ± 1 years old	CAB/AM CAB/PM CAB/BOTH CG 4 groups	Exercise: Squats, jumping jacks, running on the spot as fast as possible along with a fun story line/20 s of high-intensity activity x 10 s rest, repeated 8 times. Classroom activity breaks No drop-outs or adverse events	4 min	A single intervention	Acute	BOSS	BOSS: Positives effects for CAB/AM, CAB/PM and BOTH than CTRL.
Calvert et al. (2019) (28). Spain. RCT.	School Children *N* = 156; ♂ = 72; ♀ = 84 10–12 years old	SED: 40 LIGHT: 62 MOD: 48 VIG: 46 4 groups	One low to moderate intensity activity video (stretching and low-impact movements) and Two high intensity videos (directed fast-paced body weight exercises with jumping). ActiGraph GT3X-BT acceletometer. Exercise videos No drop-outs or adverse events	10 min	A single intervention	Acute	Toolbox Cognition Battery version 1.13. 1) DCCS 2) Flanker 3) PCT 4) PSMT	DCCS Test: SED, LIGHT, MOD and VIG: No changes in cognitive function. Flanker Test: SED, LIGHT, MOD and VIG No changes in cognitive function. PCT Test: SED, LIGHT: Significant changes in cognitive function; MOD and VIG: No changes in cognitive function. PSMT Test: SED, LIGHT, MOD y VIG: Significant changes in cognitive function; MOD and VIG: No changes in cognitive function.
Cornelius et al. (2020) (31). Unites States. RCT.	School Children *N* = 114 ♂ = 77; ♀ = 37 *M* = 16 years old	Experimental CG 2 groups	Voluntary intensity during class but moving (HR:199) Pedal desk (HR:122) No drop-outs or adverse events	>10 min	3 days per week	Study time 14 weeks. 21 days intervention or 3 waves of 2,5 weeks	Momentary time sampling (On-task behavior).	No difference between treatment and CG for the outcome of on-task behavior.
Egger et al. (2019) (33). United States. RCT.	School Children *N* = 142 ♂ = 64; ♀ = 78 *M* = 7.91 ± 0.40 years old	Combo group: 47 Aerobic group: 49 Cognition group: 46 3 groups	Combo group: The game “Horserace”: To react as quickly as possible with a predefined Movement with high cognitive engagement. Aerobic group: The game “Horserace”: To react as quickly as possible with a predefined Movement with low cognitive engagement. Cognition Group: Sat in a circle and played: the “horserace” game without any physical exertion. Using the same three keywords, the children were instructed to react as quickly as possible with their arms and fingers whenever they heard a keyword. Physical Activity Breaks No drop-outs or adverse events	10 min	5 times per week.	20 weeks.	Backwards Color Recall task (Updating). A child-adapted version of the Eriksen Flanker Task (Inhibition control). Mixed Block within the Flanker Task (Shifting).	Combo: Significant improvement compared to aerobic group. No difference compared to the cognition group in EFs (Shifting). Aerobic: Lower performance compared to combo group. No difference compared to cognition group in EFs (Shifting). Cognition: No difference compared to other two groups. Combo, Aerobic, Cognition: No significant increase in children’s shifting performance in EFs (Update). Combo, Aerobic, Cognition: No significant changes in EFs (Inhibition control).
Egger et al. (2018) (32). United States. RCT.	School Children *N* = 226 *M* = 7.94 ± 0.44 years old	Combo group high PE low PE Cognition group high CE low CE Aerobic group: Low CE High PE CG Low CE Low PE 4 groups	*Combo group*: Played a song with three special keywords car = jump up, coin = spin around, post office = sit down. The children had to react as quickly as possible to the word in song. (HR:139 ± 15.5) *Aerobic group*: Played a song with three special keywords car = jump up, coin = spin around, post office = sit down but copying the movements to the investigator. (HR:143 ± 18.4) *Cognition group*: sat in a circle and listened a song. Children were instructed to react as quickly as possible with their arms and fingers whenever they heard one out of three keywords. (HR:103 ± 8.3) *Control group:* Children sat comfortably in a circle and listened to an age-appropriate audio book for 20 min (HR:94 ± 14.6) Physical Activity Breaks (4 different plays) No drop-outs or adverse events	3× 6 min and 20 min control.	A single intervention for all groups	Acute.	Backward Color Recall task (updating). A child-adapted Eriksen Flanker task (Inhibition control). Mixed Block within the Flanker Task (Shifting).	Lower shifting performance for the children in the high CE conditions compared to the low CE conditions. No significant effects were found either for updating or for inhibition control. No significant effects were observed in any of the three core EFs or the interaction of CE and PE.
Graham et al. (2021) (27). Canada. RCT.	School Children *N* = 116 ♂ = 58; ♀ = 58 *M* = 12.19 ± 0.93 years old	Classroom-based physical activity breaks: - TPAB - APAB Sedentary classroom work 3 groups	TPAB: 50 Fitness Activity cards with predetermined exercise. HR:60–80% APAB: 50 Fitness Activity cards and solve math problems (the result is the amount of repetitions). HR:60–80% SCW: Math work at their desk Physical Activity Breaks No drop-outs or adverse events	5, 10, or 20 min	A single intervention.	Acute	Stroop task (Inhibition control). TMT (Switching). The Forward Memory Span (Updating). The Leger 20-m Shuttle Run test (Aerobic Fitness). Standing long jump.	Stroop task was greater in physical activity conditions vs. sedentary control conditions. TMT decreased significantly (participants performed better) in physical activity conditions. No significant differences in sedentary control conditions. FMS test increased significantly in the physical activity conditions- No significant differences in the sedentary control conditions. No significant main effects for condition and SLJ distance. No significant effects for physical condition by it self. But the results suggest that both aerobic and musculoskeletal fitness influence in EFs.
Howie et al. (2015) (34). United States. RCT.	School Children *N* = 96 9–12 years old	4 acute IGs performed all the treatment 4 groups	Exercise breaks (Brain BITES) intervention moderate-to-vigorous aerobic activity (marching with arm, movements and various forms of jumping and running in place reaching HR-150 bpm). Brain bites No drop-outs or adverse events	5 min, 10 min, or 20 min	A single intervention	Acute	TMT (cognitive flexibility). Digit Recall.	No improvements in executive function tasks. After 5 min: Lower aerobic fitness and greater BMI → Lower digit recall. After 20 min: Lower BMI → Greater digit recall scores
Innerd et al. (2019) (35). United States. CT.	School Children *N* = 152 ♂ = 76; ♀ = 76 *M* = 10 ± 0.7 years old	EG 72 CG: 80 2 groups	“Jumping Maths” Teacher called students to answer math problems performing number of jumps. ExCiTE No drop-outs or adverse events	10 min	3 times per week.	Study time 8 weeks. (24 interventions)	British National Coaching Foundation protocol, Shuttle Run test (Aerobic fitness) Eurofit program (Physical Fitness)	No differences in any of the components of physical fitness between CG and EG.
Kvalø et al. (2017) (37). Norway. RCT.	School Children *N* = 449 ♂ = 230; ♀ = 219 9–10 years old	IG CG 2 groups	Intervention: 2 × 45 min physically active academic lessons, 5 × 10 min physically active breaks, and 5 × 10 min physically active homework. Control: Mandatory physical education (2×45 min). Physically active homework (45 min). No drop-outs or adverse events	All the physical activities were performed during all classes or recesses (≥ 10 min).	5 time per week.	Study 40 weeks. (6 week intervention)	10-min interval running test (Aerobic Fitness). Stroop, verbal fluency, digit span, and Trail Making test (Cognitive Function). Weight, height, waist, circumference and BMI (Anthropometry).	No effects on aerobic fitness were found. Larger improvement in EFs for children IG than CG. No significant effect on BMI and waist circumference was found.
Latino et al. (2023) (28). Italy. CT.	School Children *N* = 100 ♂ = 56; ♀ = 44 *M* = 14.64 = ±0.47 years old	EG CG 2 groups	1) Warm-up: marching in place, walking jacks, knee to chest, heel digs, arm circles, shoulder rolls, knee lifts, butt kicks, lunges, side steps and high knees. 2) Main part of moderate-to-vigorous aerobic exercise 5 < RPE < 8 and (15 min). 3) Cool-down: static exercises, such as neck stretch, behind-head tricep stretch, standing hip rotation, hamstring stretch, hip flexor stretch, side stretch and butterfly stretch exercises. CG: Regular science lesson. Classroom ABs No drop-outs or adverse events	20 min	2 times per week.	Study 12 weeks. (24 sessions)	Standing long jump test, Harvard step test, push up, sit and reach test (Motor tests). BMI (Anthropometry).	Significant effect for EG in 4 physical motor tests. CG did not report any significant changes. EG showed an important decrease in BMI from pre- to post-test. CG did not report any significant decrease in BMI from pre- to post-test.
Ludyga et al. (2019) (40). Switzerland. RCT.	School Children *N* = 36 ♂ = 23; ♀ = 13 12–15 years old	EX: 20 CON:16 2 groups	EX: Aerobic and coordinative exercise session (ball games, relay games and playing tag). (HR 134.6 ± 9.4 beats/min) CON: Was encouraged to have conversations with their classmates while EX were performing ABs. Exercise session No drop-outs or adverse events	20 min	5 times per week.	8 weeks. (40 sessions)	Stroop Color-Word task (Inhibition control).	Greater reduction of reaction time on the Stroop task was observed in EX relative to CON.
Ma et al. (2015) (15). Canada. Repeated Cross-Over.	School Children *N* = 88 ♂ = 44; ♀ = 44 9–11 years old	FUNterval intervention: 53 No activity break group: 35 2 groups	Two single FUNtervals” 4-min, high-intensity interval activities using whole-body actions to complement a story-line by group. Control: FUNtervals Activity No drop-outs or adverse events	4 min	A single intervention each group	3 weeks. (week 1 familiarization, week 2 and 3 intervention)	BOSS (Off task behavior). D2 test (EFs).	Neither baseline passive or motor off-task behaviors predict the change in D2 test outcomes. A weak relationship was observed for verbal off-task behavior and improvements in D2 test performance. Students made fewer errors during the D2 test following FUNtervals.
Ma et al. (2014) (14). Canada. Repeated Cross-Over.	School Children *N* = 44 ♂ = 25; ♀ = 19 Grade 2 and 4 (no age informed)	FUNterval intervention: No activity break group: 2 groups	FUNterval: 20 s of high-intensity activity (squats, jumping jacks, scissor kicks, jumping, and running on the spot) whole body as fast as possible, separated by 10 s of rest, repeated 8 times, during story lines. No activity breaks: 10 min (lecture). FUNtervals HIIT Activity No drop-outs or adverse events	4 min	3 times per week (alternated days).	3 weeks. (9 sessions)	BOSS (off task behavior).	Improved in decreasing off-task behavior for FUNterval intervention compared to No activity break group in both levels (2° and 4° students).
Masini et al. (2023) (39). Italy. CT.	School Children *N* = 153 *M* = 7.61 ± 1.41 ♂ = 99; ♀ = 54 *M* = 7.61 ± 1.41 years old.	ABsG: CG: 2 groups	ABsG: 10 min (warm-up part of 2 min on cardiorespiratory and mobility exercises, 5 min HIIT, consisting of 40 s of MVPA alternated with 20 s of recovery, with a specific focus on coordination, balance and cognitive task. During the last 3 cool-down minutes, children performed stretching, relaxation and breathing control exercises). ActiGraph GT3X acceletometer CG: Normal lessons ABs No drop-outs or adverse events	10 min	3 times per school day. 5 times per week.	1 year and a half.	BMI (Anthropometry). The working memory cognitive test. 6 min walking test. Standing long jump test.	% of children in the normal weight category in the ABsG increased and % of children with normal weight in the CG decreased. Working memory significantly increased in the ABsG than in CG. The 6 min Cooper test increased in the ABsG but not in CG Children in the ABsG and CG significantly improved their performance in standing long jump test. Children improved their time on task behaviors in ABsG.
Mavilidi et al. (2021) (41). Australia. RCT.	School Students *N* = 221 16–18 years old	B2L Wait-list control 2 groups	B2L: Gym-HIIT- combination of aerobic (e.g., skipping) and strength exercises (e.g., squat jumps), or Sport-HIIT- using sports equipment (e.g., shuttle run while dribbling a basketball), or Class-HIIT- exercises that can be performed in a standard classroom (e.g., running on the spot, triceps dips) or Quick-HIIT- using Tabata protocol (e.g., 20 s intense work, followed by 10 s rest). Equal or more than 85% HR max. Wait-list control: continued with usual school practice. ABs No drop-outs or adverse events	10 min	2 times per week.	12 weeks. (24 sessions)	BOSS test and Applied Behavior Analysis for Teachers (On-task behavior).	Significant group-by-time effects were observed for students’ on-task behavior in favor of the B2L.
Mazzoli et al. (2021) (36). United States. RCT.	School Children *N* = 141 ♂ = 76; ♀ = 65 6–9 years old	Engaging ABs Simple ABs CG: 3 groups	Simple AB (simple imitation movement sequence) or cognitively engaging ABs (coordination sequence). ABs No drop-outs or adverse events	4–5 min	2 times per day (no specify times per week).	6 weeks.	Systematic Classroom Observations (On-task Behavior). A Go/No-Go task (Inhibition control). Toolbox List Sorting Working Memory Test (Working memory).	Showed that Simple ABs and Engaging ABs did not affect children’s cognitive functions compared to CG. Intervention had positive effects on response inhibition via a reduction in sitting time and/or an increase in standing time. No changes in working memory in any EG compared to CG.
Muñoz-Parreño et al. (2021) (30) Spain. CT.	School Children *N* = 166 ♂ = 47; ♀ = 36 *M* = 10.9 ± 0.70 years old	EG: 83 CG 83 2 groups	Two sessions of HIIT (squats, burpees, push- ups and so on) + CC, a HIIT + EI session and a HIIT + CC + CM session were conducted each day. ABs No drop-outs or adverse events	5–10 min	3–5 times per week.	17 weeks. (51–85 sessions)	NIH-EXAMINER battery: working, memory, inhibition control, cognitive flexibility.	Significant differences were produced in favor of the EG in all the variables used for the evaluation of EFs, except the continuous performance test (inhibition).
Norris et al. (2018) (43) England. RCT.	School Children *N* = 219 8–9 years old	VT: 113 CG: 106 2 groups	VT: 3 × 10 min physically active (movements of moderate-to-vigorous intensity as they “traveled” to and interacted with locations). Physically active virtual field trips. No drop-outs or adverse events	10 min	3 times per week.	6 weeks. (18 weeks intervention)	Observing Teachers and Pupils in Classrooms (OPTIC) (On-task Behavior). ACTi graph GT1M accelerometers (Physical activity).	Higher on-task behavior in the VT compared with COM group. Significant differences in PA in first two weeks but not in the last two weeks (week 3 and 4) for VT compared to CG.
Robinson K et al. (2022) (44) England. RCT.	School Students *N* = 97 ♂ = 52; ♀ = 45 *M* = 15.78 ± 0.44 years old	RTCT: 24 RTNC: 29 SECT: 21 CG: 23 4 groups	RTCT: Upper body: chair triceps dips, Namaste upper body isometric contraction, table push ups; Core: straight leg cross overs, straight leg hold, seated heel/toe taps; Lower body: Chair squats, calf raises, lunges and static hold squat + cognitive training. (RPE) RTNC: Only exercise (descripted before). SECT: Cognitive training. CON: No classwork completed for 6–8 min. Classroom ABs No drop-outs or adverse events	6–8 min	3 times per week.	4 weeks. (12 sessions)	Toolbox Cognition Battery. Flanker (Inhibitory control). Attention Test (Cognitive flexibility). BOSS (On-task behavior). 90° push-up test, 30 s maximal repetition squat to chair test and the plank hold test (Muscular fitness).	Memory improved significantly in RTNC There were no (group-by-time) effects for inhibition control or cognitive flexibility. RTCT and RTNC both improved participants’ on-task behavior in comparison with SECT and CON. Significant effects were shown for SECT on 90° push ups test compared to RTNC. CON showed better results compared to RTNC in core muscular fitness as measured using the plank hold test. No significant findings for lower body muscular endurance between groups.
Ruiz-Ariza et al. (2022) (29) Spain. CT.	School Students *N* = 162 *M* = 12.27 ± 0.47 years old	EG: 78 CG: 84 2 groups	C-HIIT program (Cognitive + moderate-high intensity intervention) 30 s work x 30 s rest x 4 reps (During the exercise they must pass a little ball to the classmate indicated by the teacher) maintained above 64% HR max. ABs No drop-outs or adverse events	4 min	5 days per week/4 times per day.	8 weeks.	D2 Test One-Minute *Ad Hoc* Test (Working memory).	No significant changes observed in memory.
van den Berg et al. (2019) (45) Netherland. RCT.	School Children *N* = 512 12 years old	EG CG 2 groups	“Just Dance”: Moderate to vigorous intensity (60% HR max). CG: Nine educational lessons, lasting 10–15 min Exercise break No drop-outs or adverse events	10 min	5 times per week.	9 weeks. (45 sessions)	D2 Test (Executive function). Stroop Color-Word task (Inhibition control). Attention Network Task (Executive control). Shuttle Run test (Aerobic fitness).	No significant differences between the EG and CG in any of the cognitive outcomes. No significant differences on aerobic fitness
Zask et al. (2023) (42) Australia. RCT.	School Children *N* = 190 5–12 years old	EG: 101 CG: 89 2 groups	Running, jumping, circuit training and others moderate to vigorous intensity options, such as games, for example, “bull-rush” and “sticking” in the mud.” ABs No drop-outs or adverse events	10 min	5 times per week (3 intervention per day).	6 weeks. (90 sessions)	TMT (EFs).	Significantly fewer children engaged in off-task behaviors in the EG during the final observation’s week. EG and CG improved executive function. No significant differences for cognitive functioning were found when comparing the time-one to time-two differences of control and intervention students.

ABs, Active Breaks; APAB, Academic Physical Activity Break; B2L, Burn 2 Learn; BOSS, Behavioral Observation of Students in Schools; BOSS AM, Behavioral Observation of Students in Schools Morning; BOSS PM, Behavioral Observation of Students in Schools Afternoon; BOTH; CAB/AM, Classroom Physical Active-break morning; CAM/AM, Classroom Physical Active-break morning; CAM/PM, Classroom Physical Active-break morning; CC, Curriculum Content; CE, Cognitive Engagement; CG/CON, Control Group; DCCT, Dimensional Change Card Sort Test; EFs, Executive functions; EG, Experimental group; Flanker, Flanker Inhibitory Control and Attention Test; High Intensity Interval Training; M, Media; min, minutes; MNR-CBA, Mixed-methods, Non-Randomized, Exploratory Controlled Before-and-After Study; PCT, Pattern Comparison Processing Speed Test; PE, Physical Exertion; PSMT, Picture Sequence Memory Test; RCT, Randomized Control Trial; RPE, Rate of Perceived Exertion; RTNC, Resistance Training with No Cognitive Training; RTWC, Resistance Training with Cognitive Training; sec, seconds; SCNC, Sedentary Control with No Cognitive Training; SCWC, Sedentary Control with Cognitive Training; TMT, Trail Making Test; TPAB, Traditional Physical Activity Break; WSD, Within Subject Design.

### 3.1 Studies description

All included studies evaluated the effects of an intervention with ABs. A summary of the included studies is provided in Table 1. The present review found that the number of participants in each study ranged from 35 to 512 (total participants: 3,615), ranging in age from 5 to 18 years.

These studies were conducted in various countries, including Canada (15, 16, 26, 27), Spain (28–30), the United States (31–36), Norway (37), Italy (38, 39), Switzerland (40), Australia (41, 42), England (43, 44) and the Netherlands (45). Fifteen studies were RCTs (26–28, 31–34, 36, 37, 40–45), five were CTs (29, 30, 35, 38, 39), and two were repeated crossovers (15, 16).

The studies included between two and four intervention groups: 14 studies used two groups (15, 16, 29–31, 35, 37–43, 45), five studies used four groups (26, 28, 32, 34, 44), and three studies used three groups (27, 33, 36). The studies used different modalities in the application of their interventions. Six studies performed ABs based on active games (27, 32, 33, 35–37), seven studies used moderate to high-intensity physical activity (26, 29, 31, 34, 38, 42, 45), three studies applied activities based on high-intensity interval training (30, 39, 41), two studies implemented activities based on active videos (28, 43), one studies used coordination activities (40), two studies used FUNterval activities (15, 16); and one study applied resistance exercise (44). In eight studies (27, 29, 31, 32, 34, 40, 41, 45), the intensity of the activities was monitored using heart rate; two studies used the Rate of Perceived Exertion (38, 44), two studies used accelerometers (28, 39), and 10 studies did not report intervention intensity (15, 16, 26, 30, 33, 35–37, 42, 43).

The duration of the ABs varied between 3 and 20 min. Thirteen studies applied ABs for 10 min (27, 28, 30, 31, 33–35, 37, 39, 41–43, 45), five studies performed 4 min (15, 16, 26, 29, 36), two studies used ABs for 20 min (38, 40), and two studies applied 6 min (32, 44). The duration of chronic interventions in the included studies lasted from 3 weeks (15, 16) to 78 weeks (39). One study 4 weeks (44); three studies 6 weeks (36, 42, 43); three studies 8 weeks (29, 35, 40); one study lasted 9 weeks (45); two studies lasted 12 weeks (38, 41); one study lasted 14 weeks (31); one study lasted 17 weeks (30); one study lasted 20 weeks (33); and one study lasted 40 weeks (37). Five studies described acute sessions with ABs (26–28, 32, 34).

Exercise training sessions were conducted twice a week in three studies (36, 38, 41), three times a week in six studies (16, 30, 31, 35, 43, 44), five times a week in seven studies (29, 33, 37, 39, 40, 42, 45), and one intervention per group was reported in six study (15, 26–28, 32, 34).

The evaluations used in the studies focused on three variables: classroom behavior, executive function, and physical fitness. For classroom behavior, the Behavioral Observation of Students in Schools (BOSS) (15, 16, 26, 41, 44), A Go/No-Go task (36) Momentary Time Sampling test (31), Systematic Classroom Observations test (36), and Observing Teachers and Pupils in Classrooms (OPTIC) (43) were used. For executive function, the Flanker Task (28, 32, 33, 44), Trail Making Test (TMT) (27, 34, 37, 42), Stroop color-word Task (27, 40, 45), Stroop Golden Color Word (37), Attention Test (44), Attention Network Task (45), D2 test (15, 29, 45), Working Memory Cognitive Test (39), Backward Color Recall task (32, 33), Digit Span (37) The Forward Memory Span (27), Toolbox List Sorting Working Memory Test (36), Digit Recall (34) One-Minute *Ad Hoc* Test (29), Toolbox Cognition Battery (28, 44), and NIH-EXAMINER battery (30) were used. For physical fitness, Standing Long Jump (27, 39), the Shuttle Run Test (27, 35, 45), 10-min interval running test (37), 6 min walking test (39), Eurofit program (35), Motor test (38), 90° push-up test, 30 s Maximal Repetition Squat to Chair test, plank-hold test (44), and body mass index (BMI) (37–39) were used.

The results of the AB interventions are grouped as follows:

1. Classroom behavior: Positive effects were shown with the application of ABs (15, 16, 26, 41, 43, 44), and one study did not show significant differences (31).
2. Executive functions: (A) Inhibitory control showed improvements (27, 30, 36, 40), but four studies did not show significant effects (28, 32, 33, 45). (B) Cognitive flexibility: Three studies showed increases (27, 30, 44), but four did not show improvement (28, 34, 42, 45). (C) Working memory: AB interventions showed improvements (30, 39, 44), but two studies did not report an increase (29, 36).
3. Physical fitness: (A) Muscular fitness improved with AB interventions (38, 39), whereas three studies did not show an increase (27, 35, 44). (B) Cardiorespiratory fitness improved in two studies (38, 39), whereas four studies did not report a significant increase (27, 35, 37, 45). (C) Anthropometric outcomes showed improvements in AB interventions (38, 39), with only one study not showing improvement (37).

Following the descriptive analysis, we assessed the risk of bias in each study (Table 2). Study quality was assessed using the PEDro Scale (46). According to the PEDro scale criteria, the studies were categorized as follows: Criteria 1 (eligibility criteria were specified) (29, 33, 36–40, 45); Criteria 2 (subjects were randomly allocated to groups) (28, 32–34, 36–38, 40, 41, 43–45); Criteria 3 (allocation was concealed) (41, 45); Criteria 4 (the groups were similar at baseline regarding the most important prognostic indicators) (27–29, 31–45); Criteria 5 (there was blinding of all subjects) (45); Criteria 6 (there was blinding of all therapists who administered the therapy) (45); Criteria 7 (there was blinding of all assessors who measured at least one key outcome) (33, 45); Criteria 8 (measures of at least one key outcome were obtained from more than 85% of the subjects initially allocated to groups) (29, 34–41, 43–45); Criteria 9 (all subjects for whom outcome measures were available received the treatment or control condition as allocated or, where this was not the case, data for at least one key outcome were analyzed by “intention to treat”) (28, 38, 41, 44, 45); Criteria 10 (the results of between-group statistical comparisons are reported for at least one key outcome) (26–45); Criteria 11 (the study provides both point measures and measures of variability for at least one key outcome) (26–45). The overall results were: Five studies were scored as “Poor Quality” (26, 27, 31, 38, 42), 11 articles were scored as “Fair” (28, 29, 32–37, 39, 40, 43), three studies were categorized as “Good” (38, 41, 44), and one article was “Excellent” (45). Two articles were assessed using RoBANS, presenting a high quality and low risk of bias (15, 16). None of the articles in this review reported dropouts or adverse events.

**Table 2:** Assessment of risk of bias/PEDRo scale.

Study	C1	C2	C3	C4	C5	C6	C7	C8	C9	C10	C11	Score	Quality
Broad A et al. (2021) (26)	X									X	X	2	Poor
Cornelius C et al. (2020) (31)				X						X	X	3	Poor
Graham J et al. (2021) (27)				X						X	X	3	Poor
Muñoz-Parreño J et al. (2021) (30)										X	X	2	Poor
Zask A et al. (2023) (42)				X						X	X	3	Poor
Egger F et al. (2018) (32)		X		X						X	X	4	Fair
Mazzoli E et al. (2021) (36)	X	X		X				X		X	X	5	Fair
Calvert G et al. (2019) (28)		X		X					X	X	X	5	Fair
Howie E et al. (2015) (34)		X		X				X		X	X	5	Fair
Innerd A et al. (2019) (35)				X				X	X	X	X	5	Fair
Masini A et al. (2023) (39)	X			X				X		X	X	4	Fair
Ruiz-Ariza A et al. (2022) (29)	X			X				X		X	X	4	Fair
Norris E et al. (2018) (43)		X		X				X		X	X	5	Fair
Egger F et al. (2019) (33)	X	X		X			X			X	X	5	Fair
Kvalø S et al. (2017) (37)	X	X		X				X		X	X	5	Fair
Ludyga S et al. (2019) (40)	X	X		X				X		X	X	5	Fair
Mavilidi M et al. (2021) (41)		X	X	X				X	X	X	X	7	Good
Latino F et al. (2023) (28)	X	X		X				X	X	X	X	6	Good
Robinson K et al. (2022) (44)		X		X				X	X	X	X	6	Good
van den Berg V et al. (2019) (45)	X	X	X	X	X	X	X	X	X	X	X	10	Excellent

## 4 Discussion

This systematic review aimed to evaluate the effects of school-based PA interventions based on ABs on classroom behavior, executive function, and physical fitness in children and adolescent students. This review expands the evidence from recent reviews by addressing the effect of ABs conducted only in a classroom setting on classroom behavior, executive functions, and physical fitness, in contrast to previous studies (12, 17, 47). A systematic literature search found 22 studies that assessed the effects of AB interventions on classroom behavior, executive function, and physical fitness in children and adolescent students. Discussions were conducted according to the three outcomes of the study.

### 4.1 Classroom behavior

Regarding the effects of ABs on classroom behavior, six articles showed positive effects of the application of ABs. This aligns with other systematic reviews (3, 12), showing that it enhances the time spent on tasks. However, one study showed no significant differences, which contradicts findings suggesting that teachers can enhance post-break time-on-task for students with ABs in the classroom, particularly among those who are the most off-task (48). Studies that performed moderate-to-vigorous-intensity activities reported better on-task behavior, which is consistent with a study that demonstrated higher intensities of PA have a positive effect on children’s behavior (49).

### 4.2 Executive function

In relation to executive functions regarding inhibitory control, our results were unclear because four studies showed improvement, while the other four articles did not show a significant effect. This result is in the same direction as a meta-analysis that analyzed the same variables (17). Nevertheless, another experimental study reported significant effects on a specific subject (50, 51). Regarding working memory, the AB intervention showed improvements in three studies, which agrees with research on the significant effects of ABs on working memory in children (30, 52). However, other studies do not show an increase, although physical activity positively affects executive functions (53), especially working memory (2). Some studies still show that the effects are not clear, depending on the type and time of intervention (54). Our results are inconclusive about the effects of ABs on executive function, which is consistent with other similar studies (3, 12), possibly due to the lack of integration of ABs as a key aspect of learning and cognitive engagement (4).

### 4.3 Physical fitness

Regarding physical fitness, muscular fitness improved with AB intervention in two articles; this result aligns with other studies (55, 56). However, three studies did not show increases despite evidence showing improvements in muscle fitness. Regarding CRF, two studies showed improvement, while the other four studies did not report significant increases, which is unclear and does not agree with the current evidence (56, 57). Intervention with ABs improved anthropometric outcomes in two studies. These findings do not coincide with those of other studies (57, 58), but do coincide with the results of a study that did not report any improvements. One study found positive effects on flexibility, balance, and speed (35), consistent with another study; however, the evidence is limited (58). Overall, these results are unclear, which agrees with other studies conducted in the same population (59, 60).

However, the diversity of modalities reported by this study to evaluate the variables analyzed does not allow a clear discussion. Therefore, this study opens a new area of research, as homogeneity in the measurement approach is crucial for determining the effect of an intervention (9). Furthermore, based on the quality analysis of the studies, most are of poor or fair quality, which limits the robustness of the results. This is a key point to address in order to contribute to the collective construction of knowledge in the field of ABs within the school environment.

The present study showed that interventions with ABs for 4–10 min three times a week for 3 weeks or more, improved classroom behavior, while interventions for 1 year or more improved physical fitness. These results suggest that long-term ABs may improve attention and concentration during the academic task and, furthermore, contribute to reduction in cardiometabolic risk factors (17, 57). In this regard, according to the findings of this systematic review, North America (10 studies) and Europe (10 studies) are the regions where the most studies on this topic have been conducted. Therefore, the benefits of incorporation ABs would likely be most pronounced in these child population. On the other hand, the effects of ABs interventions on executive function are unclear. The incorporation of PA during school hours in children and adolescents is very scarce; therefore, promoting PA in school settings is essential (11). In this sense, ABs in our study did not show interventions with adverse effects; thus, it continues to be a viable, evidence-based formula to be incorporated into the school day (3) because currently, the school setting is considered the privileged context for children to acquire knowledge of PA habits for an integral educational process (13). The outcomes of this systematic review are important because they indicate the potential for the practical application of ABs in the school context, which can become a tool for teachers of different subjects when incorporating PA into the learning process (14).

## 5 Limitations

The limitations of this study are related to the variability in the study measures used for classroom behavior, executive functions, and physical fitness, which made it difficult to analyze the effects of ABs. Additionally, differences in the number of participants between the studies and the various modalities of AB application limited the analysis of the results. Furthermore, the quality of the studies was poor or fair; therefore, the findings should be interpreted with caution.

## 6 Conclusion

Our study provides evidence suggesting that the incorporation of ABs during school hours can improve classroom behavior in children and adolescents. These findings have important implications, especially for those seeking time-efficient PA designs aimed at improving the learning process. However, the effects of ABs on EFs and physical fitness remain unclear. According to the results, more research is needed to clarify all the effects related to the implementation of ABs programs and determine the benefits of incorporating ABs into the classroom. Lastly, it is recommended that future studies provide more detailed information about the interventions and focus on quality criteria. Additionally, it is suggested to develop a common protocol for the implementation of ABs and the measurement of their effects.

## Data Availability

The original contributions presented in the study are included in the article/Supplementary material, further inquiries can be directed to the corresponding author.
