# Supplementary material for: Effectiveness of school-based active breaks on classroom behavior, executive functions and physical fitness in children and adolescent: a systematic review
Source: Front Public Health. 2025 Jan 30;13:1469998. doi: 10.3389/fpubh.2025.1469998 (PMC11822862; doi:10.3389/fpubh.2025.1469998)
Supplement: Supplementary file 1 [file Supplementary_file_1.docx]

**Supplementary File:** **Concept map of terms for search strategy**

| **Concept 1** | **AND** | **Concept 2** | **AND** | **Concept 3** | **AND** | **Concept 4** | **AND** | **Concept 5** |
| --- | --- | --- | --- | --- | --- | --- | --- | --- |
| KEYWORDS & PHRASES |  | KEYWORDS & PHRASES |  | KEYWORDS & PHRASES |  | KEYWORDS & PHRASES |  | KEYWORDS & PHRASES |
| Active breaks |  | Students |  | Physical fitness |  | Classroom behavior |  | Executive function |
| SUBJECT HEADINGS |  | SUBJECT HEADINGS |  | SUBJECT HEADINGS |  | SUBJECT HEADINGS |  | SUBJECT HEADINGS |
| active breaks  activity break  brain break  classroom break |  | children  child  adolescent  schoolchildren  students |  | physical conditioning  cardiorespiratory fitness  muscular fitness |  | classroom behaviour  on-task behavior  off-task behavior  time-on-task |  | cognitive function  executive function  executive control  working memory  inhibitory control  cognitive flexibility |
